# Influencing factors of farmers’ participation in domestic waste classification: An empirical analysis based on the semi-nonparametric estimation extended model

**DOI:** 10.3389/fpsyg.2022.1000601

**Published:** 2022-12-16

**Authors:** Qianlu Sun, Huiqing Duan, Daojun Zhong

**Affiliations:** School of Economics and Management, Zhoukou Normal University, Zhoukou, China

**Keywords:** domestic waste classification, exemplary behavior, social supervision, consistency of willingness and behavior, semi-non-parametric estimation extended model

## Abstract

Farmers are the main participants of domestic waste classification, and their willingness and behavior to participate are directly related to the success or failure of domestic waste classification and the construction of “beautiful countryside.” Based on the analysis of the influence mechanism of exemplary behavior and social supervision on farmers’ participation willingness and behavior, an empirical analysis of 988 survey data of farmers in Henan Province is carried out using a semi-non-parametric estimation extended model. The results show that: (1) 85.63% of farmers are willing to participate in the classification of domestic waste, but their willingness and behavior are not consistent. (2) The exemplary behavior of relatives can only increase the willingness of farmers. The exemplary behavior of neighbors and village cadres not only has a positive impact on the behavior, but also facilitates the transformation of willingness to behavior. (3) The supervision of village cadres can increase the willingness of farmers. Although the supervision of villagers and cleaners will reduce the willingness of farmers, it has a significant positive impact on the behavior of farmers. Based on the research conclusions, suggestions are made to play the leading role of village cadres, attach importance to the supervision of villagers and cleaners, broaden publicity channels and strengthen publicity to special groups, improve supporting policies and classification equipment, in order to promote the classification of rural domestic waste.

## 1. Introduction

With the continuous advancement of the construction of “beautiful countryside,” the problem of rural waste management has gradually become the focus of attention of the academic community and the public. Traditional waste disposal methods such as incineration, landfill, and open storage are one of the main causes of environmental pollution in rural areas. The implementation of domestic waste classification is an effective measure to deal with the continuous increase of rural domestic waste and the continuous aggravation of environmental pollution (Kang, 2019). The “Notice on Forwarding the Implementation Plan of the Domestic Waste Classification System of the National Development and Reform Commission and the Ministry of Housing and Urban–Rural Development” issued by the (General Office of the State Council of China in, 2017) provides clear instructions on the classification of rural domestic waste, pointing out that special treatment of rural waste should be carried out to comprehensively promote the improvement of rural human settlements (General Office of the State Council of China, 2017). The “Law of the People’s Republic of China on the Prevention and Control of Environmental Pollution by Solid Wastes” revised in 2020 further clarifies the principles of domestic waste classification, pointing out that individuals and families should perform the obligation of reducing the source of domestic waste and sorting out (Agency, 2020). The No. 1 Central Document issued by the State Council of China in 2021 pointed out that “the collection, transportation and disposal system of rural domestic waste should be improved, and the waste should be classified and reduced from the source” (Agency, 2021). It can be seen that it is urgent and significant to solve the problem of domestic waste classification in rural areas. As producers and victims of rural domestic waste, farmers have the inherent motivation and potential willingness to participate in the classification of domestic waste, but their willingness and behavior are not completely consistent. Clarifying the factors that affect farmers’ willingness and behavior to participate in the classification of domestic waste, and promoting farmers to transform their willingness into behavior is the key to doing a good job in the classification of rural domestic waste.

Scholars conducted in-depth analysis and discussion around the willingness and behavior of farmers to participate in the classification of domestic waste. In terms of willingness to participate: Khan et al. (2019) explored the willingness to classify plastic waste and found that subjective norms, awareness consequences and convenience are the key factors that affect the willingness to classify. Nguyen and Watanabe (2019) conducted an investigation on the classification of domestic waste in rural areas in Vietnam, and divided the survey samples into two categories according to whether to raise poultry and livestock. The results showed that families that raise poultry and livestock have a more positive attitude toward participating in the classification of domestic waste. Jia and Zhao (2020b) conducted a comparative study between pilot areas and non-pilot areas for the classification and treatment of rural domestic waste in Shaanxi Province, and found that farmers’ concern for the environment is directly proportional to their willingness to participate in domestic waste classification, while the age and family population of farmers have a negative impact on their willingness. Qiu (2020) analyzed the reasons why farmers participate in the classification of domestic waste and found that the farmers’ willingness is mainly affected by the individual characteristics of farmers.

In terms of participation behavior: Nxumalo et al. (2020) used random sampling to investigate the disposal of plastic waste in 109 rural households and found that most rural households did not classify and recycle plastic waste. Alhassan et al. (2020) studied the influencing factors of domestic waste classification behavior and found that gender, income, residence and monetary incentives are the main factors. From the perspective of perceived value, Ma et al. (2020) analyzed the factors that affect farmers’ behavior in domestic waste classification, and concluded that spiritual benefits have a significant positive impact on domestic waste classification, while time cost and material cost have a significant negative impact on domestic waste classification. Lin et al. (2017) believed that the maintenance frequency of waste treatment equipment and the distance between houses and waste treatment equipment are the main factors that affect farmers’ behavior in domestic waste classification. Mao et al. (2019) investigated the influencing factors of farmers’ participation behavior in domestic waste classification from the perspective of psychological perception and environmental intervention, and the results showed that strengthening environmental publicity and establishing a punishment system for environmental damage can promote farmers’ participation in domestic waste classification.

Existing studies have conducted an in-depth analysis of farmers’ willingness and behavior to participate in the classification of domestic waste, but there is still room for improvement: Firstly, in terms of research perspective, the role of exemplary behavior and informal supervision has been neglected. The social network of farmers is affected by many factors such as geographical relations, clan relations, etc., so that farmers will not only consider internal factors when making decisions, but also be affected by external factors such as exemplary behavior (Yang, 2019; Qin et al., 2022). At the same time, the complexity of the living environment of farmers also makes farmers’ behavior not only subject to laws and regulations and government supervision, but also affected by social supervision from neighbors, villagers, and non-government workers. Secondly, in terms of research ideas, the logical relationship between willingness and behavior needs to be further explored. Although some scholars have studied the willingness and behavior of farmers to participate in the classification of domestic waste, there are relatively few studies on the conditions and internal mechanisms of the transformation of willingness to behavior. Specific to the influence of exemplary behavior and informal supervision on willingness and behavior, related internal logic elaboration and empirical research are also relatively scarce. Thirdly, in terms of research method, empirical model that is closer to the actual situation can be introduced. The traditional ordered logit model only changes the critical point, and the level of the explained variable does not change. Taking into account the characteristics of the research object and research area, the model assumptions can be further relaxed to meet the actual situation. Fourthly, in terms of research area, insufficient attention has been paid to the Central Plains of China. Regarding the classification of rural domestic waste, the existing research results are mainly concentrated in the developed areas in the east and the backward areas in the west, while the relevant research on the Central Plains of China still needs to be expanded. Henan Province, as a typical representative of the Central Plains, is not only a populous province but also a large agricultural province. Solving the problem of rural domestic waste classification in this area is of extraordinary significance to the governance of rural human settlements and the construction of beautiful countryside.

Therefore, we choose Henan Province as the research area. Through a survey of 988 households in Henan Province, from the perspective of exemplary behavior and social supervision, we studied the willingness and behavior of farmers to participate in the classification of rural domestic waste, found out the key factors that affect willingness and behavior, and explore the path of transformation from willingness to behavior, so as to provide policy reference for the classification of rural domestic waste in Henan Province, and provide inspiration for other regions to deal with similar problems.

## 2. Theoretical analysis and research hypothesis

### 2.1. Exemplary behavior and classification of rural domestic waste

Exemplary behavior originated from Bandura’s social learning theory. Social learning theory believes that individuals can change their own cognition and behavior by observing, learning, and imitating the behaviors of other members of the organization. In this process, individuals will be affected by exemplary behavior (Zhang and Mai, 2020). When individuals perceive the capability of an exemplar, for self-interested purposes, they will imitate the behavior of the exemplar to improve and optimize themselves, so as to make their own cognition and behavior approach the exemplar as much as possible (Dong and Wang, 2018; Zheng et al., 2021, 2022). The social activities of farmers will be affected by social variables, which is a typical social learning process. In this process, the behavior of farmers will be affected by exemplar. In the work of classification of domestic waste, the exemplar of farmer mainly comes from their own social networks. According to the embeddedness theory, social networks will have an impact on the behavior and decision-making of individuals. Therefore, the willingness and behavior of farmers to participate in the classification of domestic waste will be affected by the social network composed of relatives, neighbors, village cadres and other complex factors (Jia and Zhao, 2020a). When farmers participate in the classification of domestic waste, if they find that the exemplary behavior in their social network can reduce environmental pollution and improve the living environment, then farmers will be more willing to participate in the classification of domestic waste due to factors such as living environment and their own health.

Based on the above analysis, we propose the following research hypotheses:

*H1a:* The exemplary behavior of neighbors has a significant positive impact on farmers’ willingness to participate in the classification of domestic waste.

*H1b:* The exemplary behavior of neighbors has a significant positive impact on farmers’ behavior to participate in the classification of domestic waste.

*H2a:* The exemplary behavior of relatives has a significant positive impact on farmers’ willingness to participate in the classification of domestic waste.

*H2b:* The exemplary behavior of relatives has a significant positive impact on farmers’ behavior to participate in the classification of domestic waste.

*H3a:* The exemplary behavior of village cadres has a significant positive impact on farmers’ willingness to participate in the classification of domestic waste.

*H3b:* The exemplary behavior of village cadres has a significant positive impact on farmers’ behavior to participate in the classification of domestic waste.

### 2.2. Social supervision and classification of rural domestic waste

Social supervision refers to the supervision of various activities by individuals and social organizations other than state power organs without legal effect (Wang, 2017). The development of rural domestic waste classification is bound to be inseparable from effective social supervision. The classification of rural domestic waste presents typical characteristics of positive externalities. The time cost, labor cost, and capital cost in the classification process need to be borne by the participants themselves, but the environmental benefits obtained after treatment are public, which easily leads to the phenomenon of “free-riding” in the classification of domestic waste in rural areas. In other words, people who do not participate in the classification of rural domestic waste can also enjoy a clean environment and fresh air. To solve the “free-riding” problem of rural domestic waste classification, in addition to adopting incentive mechanisms and punitive measures, social supervision mechanisms can also be introduced. Specifically, in order to prevent those who do not cooperate with the domestic waste classification from enjoying the benefits of a clean environment for free, village cadres, villagers, cleaners, etc. can supervise the behavior of farmers, and public opinion can be used to put pressure on the perpetrators when non-compliant domestic waste disposal behaviors are discovered. As a result, when disposing of domestic waste, farmers will inevitably consider the possible consequences of criticism and social accusations caused by littering, stacking, and incineration.

Based on the above analysis, we propose the following research hypotheses:

*H4a:* The supervision of cleaners has a significant positive impact on farmers’ willingness to participate in the classification of domestic waste.

*H4b:* The supervision of cleaners has a significant positive impact on farmers’ behavior to participate in the classification of domestic waste.

*H5a:* The supervision of villagers has a significant positive impact on farmers’ willingness to participate in the classification of domestic waste.

*H5b:* The supervision of villagers has a significant positive impact on farmers’ behavior to participate in the classification of domestic waste.

*H6a:* The supervision of village cadres has a significant positive impact on farmers’ willingness to participate in the classification of domestic waste.

*H6b:* The supervision of village cadres has a significant positive impact on farmers’ behavior to participate in the classification of domestic waste.

### 2.3. Participation willingness and participation behavior

The related theoretical results of willingness and behavior mainly include the Theory of Planned Behavior (Ajzen, 1985), the Attitude-behavior Theory (Triandis, 1980), the Theory of Reasoned Action, etc. (Fishbein, 1980). Among them, the Theory of Planned Behavior is one of the commonly used theories in measuring the transformation of willingness into behavior. This theory believes that the final behavior of an individual depends not only on Attitude and Subjective Norm, but also on Perceived Behavior Control. If there is an exemplary behavior of domestic waste classification in the area where farmers live, the farmers will have a positive attitude toward domestic waste classification based on their own interests, such as air quality and living environment. With a positive attitude, the influence of subjective norm also needs to be considered. The subjective norms of the domestic waste classification behavior of farmers mainly come from the supervision of villagers, village cadres, cleaners and other groups. The supervision behavior of such groups is the main source of social pressure for farmers when deciding whether to participate in the classification of domestic waste. Finally, perceived behavior control can also affect the willingness and behaviors of farmers and can even directly determine the final behavior of farmers. Perceived behavior control mainly includes the internal perceived control and external perceived control of farmers when making decisions (Hong et al., 2019). Among them, the internal perceived control mainly includes the personal resources, family resources, and social resources of farmers, such as age, education level, family income, whether there are civil servants and village cadres in the household, etc. When the internal perceived control is insufficient, farmers’ willingness to participate may be frustrated and cannot be transformed into behavior. Just as important as internal perceived control is external perceived control. External perceived control mainly include external conditions related to domestic waste classification, such as the availability of waste classification personnel, the availability of classification equipment, and the availability of reward and punishment mechanisms. The more favorable external conditions are, the stronger the farmers’ external perceived control, and the easier it is for them to have the willingness and behavior.

Based on the above analysis, we propose the following research hypotheses:

*H7:* The willingness of farmers to participate in the classification of domestic waste has a significant positive impact on their participation behavior.

The willingness of farmers to participate in the classification of domestic waste is mainly affected by three factors, namely: attitude, subjective norms, and perceived behavior control (Alhassan et al., 2020; Nxumalo et al., 2020). The exemplary behavior of farmer’s social network will affect the farmer’s attitude to participate in domestic waste classification (Khan et al., 2019; Nguyen and Watanabe, 2019). Social supervision carried out by individuals or groups with the help of public opinion will also affect the subjective norms of farmers. The internal perceived control composed of personal resources, family resources, social resources and other internal resources and the external perceived control composed of external objective conditions jointly determine the strength of farmers’ perceived behavior control. In addition, perceived behavior control will not only affect farmers’ willingness to participate, but may also have a direct impact on farmers’ behavior.

## 3. Data sources and sample characteristics

### 3.1. Data sources

**Figure 1:**
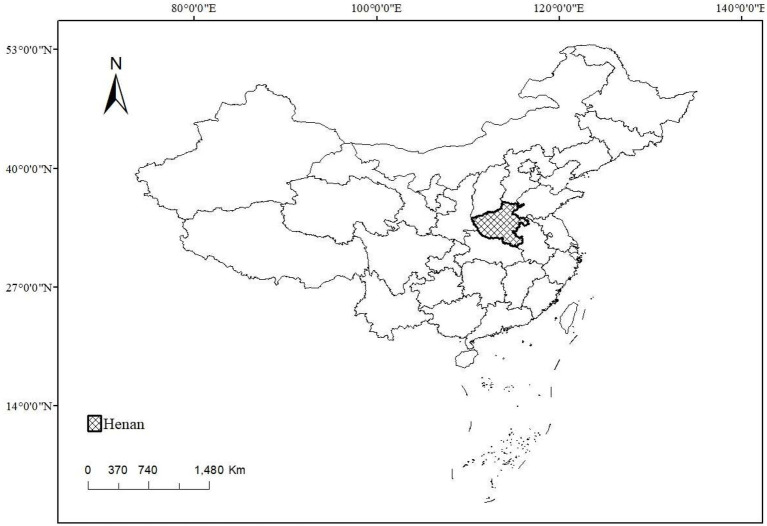
Geographical location of Henan Province on the map of China.

We choose Henan Province as the survey area. Henan Province is located in the central region of China. It is a typical populous province and a large agricultural province. The rural population of Henan province accounts for a large proportion and it is an area that cannot be ignored in the improvement of rural human settlements and rural waste management. The geographical location of Henan Province in China is shown in Figure 1.

**Figure 2:**
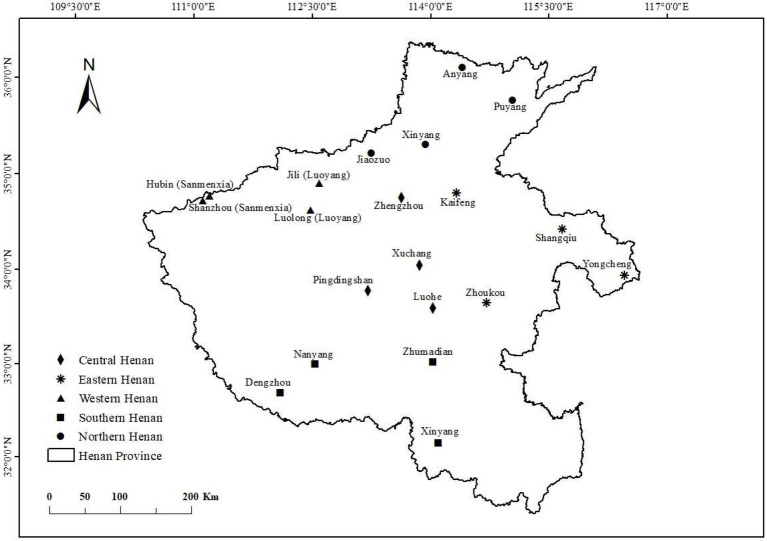
The map of sampling sites.

From January to February 2021, our research team conducted household surveys combining stratified and random sampling in five regions of Henan, including northern Henan, southern Henan, western Henan, eastern Henan, and central Henan, and randomly selected 4 sampling sites in each region. The survey content mainly includes the basic information of farmers, the willingness and behavior of domestic waste classification, and various factors that may affect domestic waste classification. A total of 1,100 questionnaires were distributed and 988 valid questionnaires were recovered through field survey. The effective rate of the questionnaire was 89.82%. A total of 87 villages were involved in the survey. Among them, 19 villages were randomly selected from the four locations of Anyang, Xinxiang, Jiaozuo and Puyang in northern Henan, and 218 farmers were surveyed. Fifteen villages were randomly selected from the four locations of Nanyang, Zhumadian, Xinyang and Dengzhou in southern Henan, and 173 farmers were surveyed. Thirteen villages were randomly selected from the four locations of Luolong (Luoyang), Jili (Luoyang), Hubin (Sanmenxia), shanzhou (Sanmenxia) in western Henan, and 164 farmers were surveyed. 22villages were randomly selected from the four locations of Kaifeng, Zhoukou, Shangqiu, Yongcheng in eastern Henan, and 231 farmers were surveyed. Eighteen villages were randomly selected from the four locations of Zhengzhou, Pingdingshan, Xuchang, Luohe in central Henan, and 202 farmers were surveyed. The map of sampling sites is shown in Figure 2.

### 3.2. Sample characteristics

In terms of sample characteristics (Table 1), the overall distribution of samples in each region is relatively balanced, and there are slightly more samples in eastern Henan. The gender ratio of the respondents was not much different, with 58.4% of males and 41.6% of females. The age distribution is dominated by the young and middle-aged groups, among which the 30 to 45-year-old samples account for the largest proportion. The education level is mainly concentrated in the junior high school and below, 41.5% of the samples have junior high school education, 40.79% of the samples have elementary school education, and the overall education level is low. In terms of income, the sample with annual household income between 9,000 and 13,000 dollars accounted for the largest proportion, accounting for 32.39%, while the sample with annual household income over 20,000 dollars accounted for the least, accounting for 9.41%. In terms of the distance from the village to the county seat, the samples within 10 km are the most, accounting for 44.33%.

**Table 1:** Sample basic characteristics.

Variable	Values	Freq.	%	Variable	Values	Freq.	%
Gender	Male	577	58.40	Age	≤30	192	19.43
	Female	411	41.60		(30,45)	313	31.68
Education	Illiterate	15	1.52		(45,60)	292	29.56
	Elementary	410	41.5		>60	191	19.33
	Middle	403	40.79	Income/10000 USD	(0,0.5)	145	14.68
	High school	125	12.65		(0.5,0.9)	193	19.53
	College and above	35	3.54		(0.9,1.3)	320	32.39
Area	Northern Henan	218	22.06		(1.3,2)	237	23.99
	Southern Henan	173	17.51		(2,+∞)	93	9.41
	Western Henan	164	16.60	Distance between village and county seat/km	(0,10)	438	44.33
	Eastern Henan	231	23.38		(10,30)	348	35.22
	Central Henan	202	20.45		(30,+∞)	202	20.45

## 4. Research methods and variable selection

### 4.1. Research methods

The willingness and behavior of farmers to participate in the domestic waste classification belong to the ordinal categorical variables. The willingness to participate can be divided into “completely unwilling,” “relatively unwilling,” “generally willing,” “relatively willing,” and “very willing.” Participation behavior can be divided into “never participated,” “rarely participated,” “frequently participated,” and “always persisted.” The values of these two variables have a strong ranking relationship, and both can be analyzed using the ordered Probit model.

The ordered Probit can estimate the parameter $\left(\beta ,r_{i}\right)$ of farmers’ willingness to participate in waste classification, but the random error item μ is required to obey the normal distribution, that is, $\mu ∼N\left(0,\delta^{2}\right)$, and it is identified by the zero intercept in the likelihood estimation through standardized processing, which is insufficient to simulate the actual situation. Hence, the estimation method of the model is extended according to Stewart’s research results considering the limitation of parameter estimation of the ordered Probit model (Stewart, 2004). Semi-nonparametric estimation, first proposed by Gallant and Nychka (1987), replaces the assumption of normal distribution of random error term μ with the Hermite form approximation density, which can be regarded as the product of a square polynomial and a normal density, thus obtaining the extended Gaussian polynomial expansion. The approximation density can be set as follows to ensure the accuracy of the approximate density:


(1) $$f_{K}\left(\varepsilon \right)=\frac{1}{\theta }{\left(\overset{K}{\underset{K=0}{\sum }}\gamma \kappa^{\varepsilon^{\kappa }}\right)}^{2}\phi \left(\varepsilon \right)$$

Where ϕ(ε) is the standard normal density function, that is,


(2) $$\theta =\overset{\infty }{\underset{-\infty }{\int }}{\left(\overset{K}{\underset{K=0}{\sum }}\gamma \kappa^{\varepsilon^{\kappa }}\right)}^{2}\phi \left(\varepsilon \right)d\varepsilon$$

It should be noted that the probability density function in the scalar multiplication vector formula is constant, so it is necessary to standardize its value, so as to obtain a set of semi-parametric distributions for the continuously increasing variable *K*:


(3) $$F_{K}\left(u\right)=\frac{\int_{-\infty }^{u}{\left(\sum_{K=0}^{K}\gamma \kappa^{\varepsilon^{\kappa }}\right)}^{2}\phi \left(\varepsilon \right)d\varepsilon }{\int_{-\infty }^{\infty }{\left(\sum_{K=0}^{K}\gamma \kappa^{\varepsilon^{\kappa }}\right)}^{2}\phi \left(\varepsilon \right)d\varepsilon }$$

It eliminates the strong oscillation of density function, and allows the difference of skewness and kurtosis, but must achieve a certain smooth condition, that is, it can approximate Hermite series with the increase of variable *K*. On the premise of satisfying this assumption, the maximum pseudo-likelihood function can be used to obtain more consistent estimation coefficients than the conventional ordered Probit model with the increase of sample size.

### 4.2. Specification of variables

There are three explanatory variables in this paper, which are Participation Willingness, Participation Behavior and Consistency of Willingness and Behavior. Likert scale was used to measure farmers’ willingness and behavior to participate in the classification of domestic waste. Regarding the measurement of consistency between willingness and behavior, the case of “being willing to participate in the classification of domestic waste but without participation behavior” was assigned to 0, and the case of “being willingness to participate in the classification of domestic waste and with participation behavior” was assigned to 1.

The main explanatory variables of this paper include two dimensions: exemplary behavior and social supervision. First of all, farmers’ willingness and behavior to participate in waste classification will be influenced by the social network composed of relatives, neighbors, village cadres and other factors (Jiang, 2020; Jia and Zhao, 2020a), which shows that the influence of exemplary behavior on farmers can be measured by the frequency of their relatives, neighbors and village cadres participating in waste classification. The influence of social supervision on the willingness and behavior of farmers can be measured from whether the villagers, the village cadres and the cleaner supervise the behavior of the farmers (Sun et al., 2020).

The control variables are divided into two categories: internal perceived control dimension and external perceived control dimension. Among them, the former mainly includes farmers’ personal resources, family resources and social resources, such as age, education level, family income, the presence of civil servants in the home, the presence of village cadres in the home, etc., the latter mainly includes external conditions related to waste classification, such as the presence of waste classification personnel, the presence of waste classification equipment, the presence of waste classification rewards and punishment measures, etc. The specific assignment and descriptive statistics of each variable are shown in Table 2.

**Table 2:** Variable value assignment and descriptive statistics.

Categories of variables	Names		Values	Mean	SE
Explained variables	Participation willingness		Completely unwilling = 1, relatively unwilling = 2, generally willing = 3, relatively willing = 4, very willing = 5	3.49	1.07
	Participation behavior		Never participated = 1, rarely participated = 2, frequently participated = 3, always persisted = 4	2.15	1.01
	Consistency of willingness and behavior		Willing but not acting =0, willing and acting =1	0.46	0.49
Key explanatory variables	Exemplary behavior	Exemplary behavior of relatives	Very few = 1, relatively few = 2, average = 3, relatively more = 4, a lot = 5	2.19	1.12
		Exemplary behavior of neighbors	Very few = 1, relatively few = 2, average = 3, relatively more = 4, a lot = 5	2.23	1.09
		Exemplary behavior of village cadres	Very few = 1, relatively few = 2, average = 3, relatively more = 4, a lot = 5	2.21	1.14
	Social supervision	Villagers’ Supervision	Never = 1, less = 2, average = 3, more = 4, a lot = 5	1.66	0.65
		Village cadres’ Supervision	Never = 1, less = 2, average = 3, more = 4, a lot = 5	1.89	1.14
		Cleaners’ Supervision	Never = 1, less = 2, average = 3, more = 4, a lot = 5	1.85	1.09
Control variables	Internal perceived control	Gender	Female = 0, male = 1	0.58	0.49
		Age	Actual age of the respondent/year-old	45.77	15.59
		Education level	Illiterate = 1, elementary = 2, junior high = 3, senior high = 4, college and above = 5	2.75	0.83
		Physical condition	Unable to work = 1, sick but can work = 2, healthy = 3	1.28	0.47
		Family income	Total income/ten thousand yuan: [0, 3] = 1, (3–5] = 2, (5–8] = 3, (8–12] = 4, (12,+∞] = 5	3.32	1.34
	External perceived control	Classification personnel	No = 0, yes = 1	0.72	0.45
		Classification equipment	No = 0, yes = 1	0.62	0.48
		Reward and punishment	No = 0, yes = 1	0.75	0.43

## 5. Results analysis and model testing

### 5.1. Analysis on consistency of willingness and behavior

According to the descriptive statistical results (Table 3) of the consistency of farmers’ willingness and behavior to participate in the classification of domestic waste, farmers had a high participation willingness, and 846 of the 988 surveyed farmers were willing to participate in, accounting for 85.63%. However, only 45.74% (387 farmers) of the respondents who with the participation willingness had the behavior of classification of domestic waste, showing significant inconsistency between their willingness and behavior, so hypothesis 7 was not been verified. Specifically, in terms of gender distribution, females’ participation willingness and consistency of willingness and behavior were higher than those of males, which were 86.37 and 49.01%, respectively. Regarding the age distribution, the highest consistency of willingness and behavior was 53.65% for the 31 to 45 years old samples. The samples with annual household income between 5,000 dollars and 9,000 dollars showed the strongest willingness to participate in domestic waste classification, with 91.73%.

**Table 3:** Descriptive statistics of consistency of willingness and behavior.

Variables	Options	Participation willingness				Consistency of willingness and behavior	
		Number of unwilling samples	%	Number of willing samples	%	Number of samples with willingness and behavior	%
Gender	Male	86	14.9	491	85.1	213	43.38
	Female	56	13.63	355	86.37	174	49.01
Age	≤30	8	4.17	184	95.83	78	42.39
	(30,45)	39	12.46	274	87.54	147	53.65
	(45,60)	44	15.07	248	84.93	96	38.71
	≥60	51	26.7	140	73.3	66	47.14
Annual household income/10000 USD	(0,0.5)	17	12.59	118	87.41	72	61.02
	(0.5,0.9)	11	8.27	122	91.73	54	44.26
	(0.9,1.3)	31	12.92	209	87.08	73	34.93
	(1.3,2)	30	12.66	207	87.34	109	52.66
	(2,+∞)	53	21.81	190	78.19	79	41.58
Total samples		142	14.37	846	85.63	387	45.74

### 5.2. Factors influencing transformation from willingness to behavior: Correlation analysis

In order to analyze the factors influencing transformation from farmers’ willingness to behavior when they participate in domestic waste classification, Pearson correlation coefficient method was used to preliminarily test the correlation between the key explanatory and control variables and the consistency of willingness and behavior. The results showed that (Table 4) variables such as exemplary behavior of relatives, exemplary behavior of neighbors, exemplary behavior of village cadres, villagers’ supervision, village cadres’ supervision and cleaners’ supervision had significant positive effects on the transformation of farmers’ willingness to participate in waste classification into behavior. Such variables as gender, age, family income, whether there is a civil servant, classification personnel, classification equipment, and incentive measures had a significant negative impact. Education level, physical condition, whether there are village cadres or not had weak correlation with it. Peterson correlation coefficient reflects the relationship and significance between various variables and the transformation from intention to behavior, which can give us a preliminary understanding of the influencing factors of the transformation from intention to behavior. However, whether a variable is still significant under the joint influence of each variable needs further analysis.

**Table 4:** Statistical table of Pearson correlation coefficient between key explanatory variables and control variables in farmer waste classification.

Independent variables	Correlation coefficients	Independent variables	Correlation coefficients
Exemplary behavior of relatives	0.4169^***^	Education level	0.0429
Exemplary behavior of neighbors	0.4351^***^	Physical condition	−0.0443
Exemplary behavior of village cadres	0.4168^***^	Household income	−0.1570^***^
Villagers’ supervision	0.4626^***^	Whether there is a civil servant	−0.0640^*^
Village cadres’ supervision	0.3596^***^	Whether there is a village cadre	−0.0077
Cleaners’ supervision	0.3735^***^	Classification personnel	−0.3221^***^
Gender	−0.1287^***^	Classification equipment	−0.3915^***^
Age	−0.1438^***^	Reward and punishment mechanisms	−0.3331^***^

*, ** and *** in the table indicate significance at the statistical levels of 10, 5 and 1%, respectively.

### 5.3. Influencing factors of willingness and behavior: An extended semi-nonparametric estimation model

To verify the research hypotheses proposed above, further analysis is needed on the effects of model behavior, social supervision and other control variables on farmers’ willingness, behavior and the consistency of willing and behavior to participate in domestic waste classification. Before model estimation, the coefficient of variance expansion (VIF) was used to test the multicollinearity among the explanatory variables, and the results showed that the maximum value of VIF was 3.65, and the mean value of VIF was 1.73, indicating that there was no obvious multicollinearity problem among the explanatory variables. According to the variable settings mentioned above, Stata16.0 software was used to fit the farmers’ willingness and behavior to participate in domestic waste classification using the semi-nonparametric estimation expansion model. The results are shown in Table 5 (Model 1 and Model 2). In order to analyze the influencing factors and internal mechanism of the transformation of farmers’ willingness to participate in waste classification into behavior, a willingness-behavior transformation model (Model 3) was set in this paper. The “very unwilling” and “relatively unwilling” in the participation willingness were considered as unwilling, and assigned to 0 in Model 3, “generally willing,” “relatively willing” and “very willing” as willing, and assigned a value of 1 in Model 3. At the same time, “never participated” and “rarely participated” in participating behaviors were regarded as no behaviors, and assigned to 0 in Model 3, “always participated” and “always persisted” as behaviors, and assigned a value of 1 in Model 3. The explained variable of the willingness behavior transformation model is a binary selection variable, which can be solved using binary logit or binary probit. However, these two methods have high requirements for parameter assumptions, so the generalized maximum entropy logit method with relaxed assumptions was used for fitting, and the results are shown in Table 5 (Model 3).

**Table 5:** Estimation results of three different models of rural residents’ willingness and behavior consistency.

Variables	Participation willingness (Model 1): Completely unwilling → very willing			Participation Behavior (Model 2): Never participated → always persisted			Willingness Transformation Behavior (Model 3): Participation willingness → participation behavior		
	Coefficients	SE	*Z*	Coefficients	SE	*Z*	Coefficients	SE	*Z*
Exemplary behavior of Relatives	0.1947^***^	0.0752	2.59	0.1502	0.0935	1.61	−0.0823	0.1731	−0.48
Exemplary behavior of Neighbors	0.0733	0.0683	1.07	0.2816^***^	0.0872	3.23	0.3241^**^	0.1492	2.17
Exemplary behavior of village cadres	0.1007	0.0668	1.51	0.1733^**^	0.0826	2.1	0.4801^***^	0.1560	3.08
Villagers’ supervision	−0.1194^*^	0.0709	−1.68	0.5470^***^	0.0904	6.05	0.5128^***^	0.1615	3.18
Village cadres’ supervision	0.1563^**^	0.0615	2.54	−0.0093	0.0736	−0.13	−0.0121	0.1365	−0.09
Cleaners’ supervision	−0.1562^**^	0.0654	−2.39	0.1509^*^	0.0780	1.94	0.0996	0.1434	0.69
Gender	−0.1382^**^	0.0689	−2.01	−0.2799^***^	0.0907	−3.09	−0.5309^***^	0.1674	−3.17
Age	−0.0119^***^	0.0025	−4.76	−0.0075^**^	0.0030	−2.54	0.0060	0.0055	1.09
Education level	0.0434	0.0381	1.14	0.0387	0.0493	0.79	−0.0020	0.0988	−0.02
Household income	−0.0841^***^	0.0262	−3.21	−0.0563	0.0352	−1.6	0.0173	0.0633	0.27
Classification personnel	−0.1130	0.1226	−0.92	0.0681	0.1591	0.43	0.0855	0.3035	0.28
Classification equipment	0.2342^**^	0.1124	2.08	−0.1981	0.1415	−1.4	−0.1749	0.2603	−0.67
Reward and punishment mechanisms	0.2022^*^	0.1077	1.88	0.1453	0.1373	1.06	−0.1232	0.2624	−0.47
Overall test statistics of model	*N* = 988 Wald chi^2^(16) = 61.46 Prob>chi^2^ = 0.0000 Log likelihood = −1308.35			*N* = 988 Wald chi^2^(16) = 131.31 Prob>chi^2^ = 0.0000 Log likelihood = −1037.82			*N* = 846 Degrees of freedom = 16 Entropy for probs. = 481.7 Normalized edtropy = 0.8215 Ent.ratio stat. = 209.4 *P* Val for LR = 0.0000 Pseudo-*R*^2^ = 0.1785 Criterion F (log L) = −2340.09		

*, ** and *** in the table indicate significance at the statistical levels of 10, 5 and 1%, respectively. In the table, Degrees of freedom is the degree of freedom, Entropy for probs is the probability entropy, Normalized entropy is the standard entropy, Ent. ratio stat is the statistical ratio entropy, P Val for LR is the p value in the likelihood ratio test, and CriterionF is the standard F value of log-likelihood estimation.

#### 5.3.1. Key explanatory variables

Exemplary behavior. The exemplary behavior of relatives had a significant positive effect on farmers’ willingness to participate in domestic waste classification, but no significant effect on the participation behavior and consistency of willingness and behavior, indicating that the higher the frequency of relatives’ participation in domestic waste classification, the stronger the willingness of farmers to participate. However, the behavior of relatives could not effectively promote the participation behavior of farmers, nor could it achieve the transformation of willingness to behavior. Clearly, H2a was verified, but H2b was not. The exemplary behavior of neighbors and village cadres had significant positive effects on the behavior of farmers’ participation in domestic waste classification and the consistency of willingness and behavior, which indicated that the higher frequency of the neighbor and the village cadres’ participation in domestic waste classification, the more obvious the model role for farmers. Accordingly, the more frequent the farmers’ participation behavior, the higher the consistency of willingness and behavior. Thus, H1b and H3b were verified, while H1a and H3a were not.

Social supervision. Village cadres’ supervision had a significant positive effect on farmers’ willingness to participate in domestic waste classification, but had a weak effect on the participation behavior and consistency of willingness and behavior, indicating that the higher the frequency of village cadres’ supervision over domestic waste classification, the stronger the farmers’ participation willingness. However, village cadres’ supervision could not promote farmers’ participation behavior, nor can it convert participation willingness into behavior. Thus, H6a was verified, but H6b was not. The villagers’ supervision and the cleaner’s supervision had a significant negative impact on the farmers’ willingness to participate in the domestic waste classification. In other words, the more frequently the villagers and the cleaner supervised the domestic waste classification, the lower the farmers’ participation willingness. However, the villagers’ supervision and the cleaner’s supervision have a significant positive impact on the farmers’ participation behavior. The higher the supervision frequency, the more frequent the farmers’ participation behavior. Thus, H4b and H5b were verified, but H4a and H5a were not.

#### 5.3.2. Control variables

The age of the respondents had a significant negative impact on the willingness and behavior of farmers to participate in the classification of domestic waste. In terms of gender, the willingness, behavior, consistency of willingness and behavior of female samples participating in domestic waste classification were higher than those of male samples. Physical condition had a negative effect on the consistency of willingness and behavior and was significant at the statistical level of 1% and household income had a significant negative impact on farmers’ willingness. In addition, the classification equipment and rewards and punishment measures had a significant positive impact on farmers’ willingness to participate in domestic waste classification, but had no significant impact on the participation behavior and the consistency of willingness and behavior.

## 6. Discussion

Guo et al. (2017), in order to effectively control the rapid growth of domestic waste, used the waste classification plus system to improve the accuracy of waste classification and residents’ environmental awareness. The semi quantitative case study method was used to analyze the effect of the waste classification plus points system. The results show that the use of the scoring system can improve the accuracy and awareness of waste classification, thus affecting the management of domestic waste. In general, residents support and benefit from the system, which improves their participation and environmental awareness. However, continuous advocacy and policy coordination are needed to promote the widespread implementation and sustainable development of this system. In this study, we found that the reward and punishment system can effectively improve the willingness of residents to participate in household waste classification. In the research process, providing waste classification equipment for farmers and formulating corresponding reward and punishment measures can not only improve farmers’ willingness to participate in the classification of domestic waste, but also improve farmers’ behavior in the classification of domestic waste, which may be related to the poor practicability of rural waste classification equipment, unreasonable reward and punishment mechanism and other reasons. The research results of Tang et al. (2019) show that taking corresponding incentive measures can greatly improve the enthusiasm of rural residents for waste classification, thus helping to create a better living environment in rural areas. This research result once again shows that a reasonable incentive mechanism can help farmers improve their willingness to participate in household waste classification.

Jia and Zhao (2020a) studied the impact of household waste pollution perception and social capital on household waste classification, and found that farmers’ willingness and behavior to participate in waste classification were significantly related to their social network composed of relatives, village cadres, neighbors and other factors. Among them, the more positive the willingness and behavior of the social network groups around farmers to participate in waste classification, the more positive the willingness and behavior of farmers will be. In this study, the results show that the exemplary behavior of relatives can only affect farmers’ willingness to participate, but has no significant impact on farmers’ behavior and the consistency of their willingness and behavior. The exemplary behavior of neighbors and village cadres can not only promote action, but also help to transform their will into behavior, which can have a significant impact on farmers’ participation behavior and the consistency of their will and behavior. Zhang and Mai (2020) conducted an in-depth study on the impact of neighbor and surrounding example behaviors on waste classification, and found that the example behaviors around farmers can not only help them participate in waste classification more actively, but also improve farmers’ willingness to lead others to participate in waste classification. This result is also consistent with the result of this experiment, which shows that for rural people, the waste classification exemplary behavior of the surrounding social network can greatly affect them, so reasonable waste classification exemplary behavior can be used to promote the willingness and behavior of rural people to do waste classification.

Nguyen and Watanabe (2019) found that the behavior process and results of farmers’ neighbors and village cadres participating in household waste classification will directly have a significant impact on farmers. The results show that the exemplary behavior of farmers’ neighbors and village cadres can effectively promote the change of farmers’ willingness and specific behavior to participate in waste classification. This result is similar to the conclusion of this study, that is, the exemplary behavior of farmers’ neighbors and village cadres can effectively promote the behavior and willingness of farmers to participate in waste classification. In addition, this study also found that social supervision has both positive and negative effects. The supervision of village cadres can enhance their will, but has no significant impact on their behavior and the consistency of their will and behavior. Although the supervision of villagers and cleaners will reduce farmers’ willingness to participate, they can promote farmers’ participation behavior. Kang (2019) found in his research on the influencing factors of farmers’ waste classification behavior in 2019 that the stronger the willingness of rural residents’ neighbors to classify waste, the stronger the ability of rural residents to classify waste. This also verified the conclusion of this study that the exemplary behavior of farmers’ neighbors can effectively improve the waste classification awareness of rural residents. Nxumalo et al. (2020) also found in their research on waste classification in rural areas that in villages where village cadres do a good job of waste classification, the waste classification awareness and behavior of rural residents are higher than other villages, which is consistent with the results of this study. To sum up, the exemplary behavior farmers’ neighbors and village cadres plays an important role in the process of rural waste classification.

In the research of Alhassan et al. (2020), female family members pay more attention to the classification of domestic waste, and the classification behavior of domestic waste is usually dominated by female members. The study also found that female rural residents’ waste classification intentions, behaviors and consistency of intentions and behaviors were higher than those of men. As far as the age range of rural residents is concerned, the environmental awareness of young people is stronger than that of the elderly. Young rural residents have a better understanding of the hazards of unclassified waste, so they have stronger willingness to participate and more frequent participation behaviors. In addition, the study also found that public supervision has a great role in promoting the waste classification behavior and willingness of rural residents. Specifically, the greater the public supervision, the stronger the willingness and behavior of rural residents to participate in waste classification. Sun et al. (2020) found in their research on farmers’ willingness and behavior to participate in improving the rural living environment that social planning and public supervision can effectively improve farmers’ enthusiasm to participate in improving the rural living environment. The results can also support the results of this experiment. Social public supervision has a positive impact on the waste classification of rural residents. We can improve the enthusiasm of rural residents for waste classification through public supervision, so as to improve the quality of rural living environment.

## 7. Conclusion and enlightenments

### 7.1. Conclusion

Based on 988 household survey data in Henan Province, the influencing factors of farmers’ willingness and behavior to participate in domestic waste classification were analyzed using a semi-nonparametric estimation extension model from the perspective of exemplary behavior and social supervision. Then, the consistency of farmers’ willingness and behavior was discussed using the generalized maximum entropy logit model. The conclusions are as follows:

1. There is a significant inconsistency in willingness and behavior. Farmers have a high enthusiasm to participate in the classification of domestic waste, 85.63% of them are willing to participate, but only 45.74% of them are willing and had behaviors at the same time, showing a situation of “high willingness and low behavior.”
2. Different exemplary behaviors have different influences on farmers. The exemplary behavior of relatives can enhance farmers’ participation willingness, but it has no obvious influence on behavior and consistency of willingness and behavior. The exemplary behavior of neighbors and village cadres can not only boost actions, but also help to transform willingness into behavior. In other words, the higher the frequency of neighbors’ and village cadres’ participation in domestic waste classification, the more frequent the farmers’ participation behavior, and the higher the consistency of willingness and behavior.
3. Social supervision has both positive and negative effects. Supervision of village cadres can enhance willingness, but it has no obvious influence on behavior and consistency of willingness and behavior. Although the villagers’ and the cleaner’s supervision will reduce the farmers’ participation willingness, they can promote the farmers’ participation behavior. In other words, the higher the frequency of supervision of the villagers and the cleaner on the classification of domestic waste, the lower the willingness of farmers to participate, but the more frequent the participation behavior.
4. In terms of control variables, young people are more willing to participate, and their behaviors are more frequent. Women’s willingness, behavior and consistency of willingness and behavior are higher than those of men. Low-income families and sick people pay more attention to the classification of domestic waste. Providing classification equipment and reward and punishment mechanisms can enhance farmers’ participation willingness.

### 7.2. Enlightenment

The leading role of village cadres and the supervisory role of villagers and cleaning staff should be brought into play. Because village cadres are not only the administrative representatives of villages, but also the direct responsible persons of rural household waste classification, whose behavior is directly related to the effect of household waste classification, so they should take the lead in participating in domestic waste classification, perform relevant supervisory duties, and promote household waste classification. It is an effective measure to deal with the problem of “hitchhiking” by supervising the domestic waste classification by villagers and cleaners. Besides, special posts for supervision of waste classification can be set up to supervise farmers’ domestic waste disposal methods and guide farmers to develop the habits of waste classification.

Propaganda channels should be broadened, and the propaganda and exemplary behavior of special groups should be emphasized. Due to the difficulty in attracting farmers’ attention and limited penetration of traditional propaganda methods such as posting slogans and holding conferences, attention should be paid to the roles of neighbors and relatives, and propaganda methods should be diversified and channels should be broadened. For example, the concept of waste classification should be spread through the channels of neighbors and relatives by means of distributing daily necessities containing propaganda slogans, compiling jingles or songs, etc. Targeted propaganda should be made for families of low-income and seriously ill patients. Specifically, the economic benefits of domestic waste classification should be emphasized for low-income families, while the ecological benefits of domestic waste classification should be emphasized for families with seriously ill patients, so that the benefits of domestic waste classification can be spread more widely. Furthermore, women should be encouraged to lead family members to participate in domestic waste classification, and do a good job in propaganda and mobilization of young groups, which can also speed up the pace of domestic waste classification in rural areas.

The supporting policies should be improved, and classification equipment should be provided. Since the existing waste classification policies and waste classification facilities and equipment in rural areas to a certain extent increase the enthusiasm of farmers to participate, but cannot improve the behavior frequency and the consistency of willingness and behavior of farmers, the existing supporting policies can be revised and improved to adapt them to the actual situation in different rural areas, and more practical and functional classification equipment can be provided to improve the experience of farmers’ domestic waste classification.

## Data availability statement

The original contributions presented in the study are included in the article/Supplementary material, further inquiries can be directed to the corresponding author.

## Author contributions

DZ contributed to conception and design of the study, and wrote the first draft of the manuscript. QS organized the database. HD performed the statistical analysis. All authors contributed to manuscript revision, read, and approved the submitted version.

## Conflict of interest

The authors declare that the research was conducted in the absence of any commercial or financial relationships that could be construed as a potential conflict of interest.

## Publisher’s note

All claims expressed in this article are solely those of the authors and do not necessarily represent those of their affiliated organizations, or those of the publisher, the editors and the reviewers. Any product that may be evaluated in this article, or claim that may be made by its manufacturer, is not guaranteed or endorsed by the publisher.

## Supplementary material

The Supplementary material for this article can be found online at: https://www.frontiersin.org/articles/10.3389/fpsyg.2022.1000601/full#supplementary-material
