# Supplementary material for: Influencing factors of farmers’ participation in domestic waste classification: An empirical analysis based on the semi-nonparametric estimation extended model
Source: Front Psychol. 2022 Dec 16;13:1000601. doi: 10.3389/fpsyg.2022.1000601 (PMC10115184; doi:10.3389/fpsyg.2022.1000601)
Supplement: Supplementary file 1 [file Data_Sheet_1.docx]

***Appendix 1: Robustness Test***

It is necessary to test the robustness of the estimation results of the extended semi-parametric estimation model and the generalized maximum entropy logit model in order to verify the reliability of the previous regression results. In this paper, two methods were used to test the direction and significance of the estimated parameters. First, the ordered logit model was used to re-estimate the participation willingness model (model 1) and participation behavior model (model 2) with ordered dependent variables, and the results are shown in Table 6 (models 4 and 5). The binary logit model was used to re-estimate the willingness-to- behavior transformation model with dependent variables as binary variables (Model 3) and the results are shown in Table 6 (Model 6). The comparison between the previous regression results (Model 1, Model 2, Model 3) and the logit model regression results (Model 4, Model 5, Model 6) in Table 6 showed that the influence direction and significance of the key independent variables on the dependent variables had not changed significantly after the estimation method was changed. Secondly, although the control variables such as individual characteristics, internal resources and external conditions of the sample were added in the analysis of farmers’ participation willingness, participation behavior and the influencing factors of consistency of willingness and behavior, some important variables that affect the overall structure of the model may still be omitted, resulting in the result bias. Here, the two control variables of government propaganda frequency and government funding support were added, and the model was re-estimated according to the above estimation method to again verify the robustness of the above regression result. The results are shown in Appendix Table 1 (Models 7-12). According to the regression result after the adding the two control variables, the key variables that had significant effects on participation willingness, participation behavior and willingness conversion behavior in the previous results were still significant in Models 7-12. So, the research results of this paper are relatively robust.

**Appendix Table 1 Results of robustness test**

| Variables | Logit model | | | Adding government propaganda frequency | | | Adding government funding support | | |
| --- | --- | --- | --- | --- | --- | --- | --- | --- | --- |
|  | Participation willingness (model 4) | Participation behavior (model 5) | Transformation from willingness to behavior (model 6) | Participation willingness (model 7) | Participation behavior (model 8) | Transformation from willingness to behavior (model 9) | Participation willingness (model 10) | Participation behavior (model 11) | Transformation from willingness to behavior (model 12) |
| Relative model role | 0.2381^*^ (0.1358) | 0.1968 (0.1376) | -0.0826 (0.1736) | 0.1938^*^ (0.0750) | 0.1553^*^ (0.0942) | -0.0784 (0.1730) | 0.1981^***^ (0.0752) | 0.1354 (0.0932) | -0.1180 (0.1756) |
| Neighbor model role | 0.0713 (0.1212) | 0.4132^***^ (0.1225) | 0.3260^**^ (0.1496) | 0.0723 (0.0677) | 0.2858^***^ (0.0880) | 0.3210^**^ (0.1493) | 0.0747 (0.0685) | 0.2785^***^ (0.0860) | 0.3451^**^ (0.1507) |
| Village cadre model role | 0.3211^***^ (0.1234) | 0.3221^***^ (0.1234) | 0.4830^***^ (0.1565) | 0.1002 (0.0664) | 0.1709^**^ (0.0836) | 0.4775^***^ (0.1560) | 0.1070 (0.0684) | 0.1714^**^ (0.0810) | 0.4654^***^ (0.1569) |
| Villagers’ supervision | -0.3467* (0.2044) | 0.8555^***^ (0.1338) | 0.5162^***^ (0.1620) | -0.1139^*^ (0.0705) | 0.5505^***^ (0.0906) | 0.5185^***^ (0.1617) | -0.1256^*^ (0.0727) | 0.5701^***^ (0.0892) | 0.5766^***^ (0.1656) |
| Village cadre’s supervision | 0.2268^**^ (0.1078) | -0.0608 (0.1094) | -0.0122 (0.1368) | 0.1513^**^ (0.0617) | -0.0128 (0.0744) | -0.0160 (0.1368) | 0.1513^**^ (0.0619) | -0.0158 (0.0730) | -0.0193 (0.1374) |
| Cleaner’s supervision | -0.2644^**^ (0.1140) | 0.2318^**^ (0.1161) | 0.1007 (0.1438) | -0.1470^**^ (0.0666) | 0.1711^**^ (0.0811) | 0.1168 (0.1469) | -0.1462^**^ (0.0672) | 0.1556^*^ (0.0798) | 0.1056 (0.1474) |
| Government propaganda frequency | — | — | — | 0.0635 (0.1016) | 0.1510 (0.1364) | 0.1381 (0.2488) | 0.0654 (0.1030) | 0.1330 (0.1343) | 0.1406 (0.2508) |
| Government funding support | — | — | — | — | — | — | -0.0645 (0.0683) | 0.1825^**^ (0.0881) | 0.3444^**^ (0.1750) |
| Control variables | Controlled | Controlled | Controlled | Controlled | Controlled | Controlled | Controlled | Controlled | Controlled |

Note: *, * * and * * * in the table indicate the significance at statistical levels of 10%, 5% and 1%, respectively. The standard errors are in brackets. To save space, other control variables and the overall test statistics of the model have been omitted.

***Appendix 2: Questionnaire***

The research collected information about waste classification from rural residents through questionnaires. The specific contents of the questionnaire are shown in below.

1. What is your gender?

A. Male

B. Female

2. Your home address (specific to counties and cities).

3. How many people are there in your family?

4. Your education background?

5. Your age?

6. Your family's annual income?

7. The distance from your village to the nearest county?

8. How many Kg of household waste are produced in your home every week?

9. What are the main domestic wastes in your home (multiple choices are allowed, and please sort them from more to less)

A. Waste left in the kitchen

B. Recyclable items, such as waste paper, plastic bottles, metals, etc

C. Articles difficult to reuse, such as broken glass, broken ceramics, etc

D. Serious pollutants, such as waste batteries, pesticide bottles, plastic bags, agricultural films, etc

The order is:____________________

10. How does your family usually deal with household waste? (Multiple choices are allowed. Please sort them from most to least)

A. Self recycling

B. In situ incineration

C. Random dumping

D. Sold to the scrap purchase station

E. Stacked at designated place

F Others__________

The order is______________________

11. Does your family recycle and classify household waste?

A. Will

B. Occasionally

C. Never

12. What is the main purpose of your classified recycling of domestic waste?

A. Earn some expenses

B. Protect the environment

C. Village requirements

D. Recycling

13. Which domestic wastes do you usually recycle? (Multiple choices are allowed)

A. Glassware

B. Paper

C. Metal

D. Plastic bottle

E. Clothing

F. Other______________________

14. How much does the model behavior of relatives around the waste classification affect your behavior?

A. Very small

B. Relatively small

C. Medium

D. Relatively large

E. Big

15. How much does the model behavior of relatives around the waste classification affect your behavior?

A. Very small

B. Relatively small

C. Medium

D. Relatively large

E. Big

16. How much does the exemplary behavior of the surrounding village cadres on waste classification affect your behavior?

A. Very small

B. Relatively small

C. Medium

D. Relatively large

E. Big

17. How much does the exemplary behavior of the surrounding village cadres on waste classification affect your wishes?

A. Very small

B. Relatively small

C. Medium

D. Relatively large

E. Big

18. How much does the supervision of village cadres on waste classification affect your behavior?

A. Very small

B. Relatively small

C. Medium

D. Relatively large

E. Big

19. How much does the supervision of village cadres on waste classification affect your wishes?

A. Very small

B. Relatively small

C. Medium

D. Relatively large

E. Big

20. How much does the supervision of cleaners on waste classification affect your behavior?

A. Very small

B. Relatively small

C. Medium

D. Relatively large

E. Big

21. How much does the supervision of cleaners on waste classification affect your wishes?

A. Very small

B. Relatively small

C. Medium

D. Relatively large

E. Big

22. The ability to distinguish recyclable waste from non recyclable waste?

A. Very clear

B. Clear

C. Fuzziness

D. I don't know, I don't care

23. Does your village have infrastructure for centralized waste treatment?

A. Yes

B. No

C. Not clear

24. Why do you think there is no waste disposal mechanism in the village?

A. Not enough funds

B. Didn't realize the harm of waste

C. The village committee is not organized

D. Technical issues

25. How do you usually deal with household waste in your village? (Multiple choices are allowed)

A. Unified incineration

B. Centralized burial

C. Classified recycling

D. Biogas development

E. Other________________

26. Do you often see household waste in your village?

A. Yes

B. No

C. Occasionally, but someone cleans up

27. Do you know the harm of littering household waste to human beings and the environment?

A. Very familiar

B. Learn a little

C. Not at all

28. Have you ever conducted environmental protection policy publicity in your village?

A. Often

B. Occasionally

C. No

29. Do you think it is necessary to dispose the waste?

A. It is necessary

B. Not necessary

C. Dispensable

30. Why do you think it is unnecessary to dispose of waste?

A. Waste money

B. Waste can be degraded by itself

C. Don't know the harm of waste

31. Are you satisfied with the local domestic waste treatment? Do you have any better opinions or suggestions?
